# Discrete Mathematics in Dynamic Network Analysis: Long-Term Efficacy Evaluation of Fotona Laser Therapy for Overactive Bladder Syndrome Using Clustering-Based Patient Subgroup Identification

**DOI:** 10.7759/cureus.68671

**Published:** 2024-09-04

**Authors:** Nobuo Okui, Machiko Okui

**Affiliations:** 1 Dentistry, Kanagawa Dental University, Yokosuka, JPN; 2 Urogynecology, Yokosuka Urogynecology and Urology Clinic, Yokosuka, JPN

**Keywords:** precision medicine, principal component analysis, clustering analysis, fotona laser therapy, overactive bladder syndrome, dynamic network analysis, discrete mathematics

## Abstract

Dynamic network analysis, a state-of-the-art application of discrete mathematics, offers unprecedented insight into complex, time-evolving clinical data. This technical report demonstrates the value of evaluating the long-term efficacy of Fotona laser therapy (Fotona d.o.o., Ljubljana, Slovenia) in overactive bladder (OAB) syndrome. We analyzed data from 101 female patients aged ≥60 years who underwent Fotona laser treatment, including Vaginal Erbium Laser (VEL) and Urethral Erbium Laser (UEL), between 2020 and 2022. OAB symptom scores (OABSS) were collected at baseline (T0) and at six, 12, 18, and 24 months post treatment. Network graphs were constructed, representing patients as nodes, and symptom similarities as edges. Clustering techniques identify patient subgroups, whereas principal component analysis reduces dimensionality. The dynamic evolution of patient clusters was visualized through changes in average degree centrality over time. This approach revealed three distinct patient clusters with unique treatment response patterns. The results showed a progressive reduction in OABSS, with the total score decreasing by 2.82 ± 3.03 at 24 months. Our method provides novel visualization and analysis of complex longitudinal clinical data, offering insights into personalized treatment strategies for OAB. This report presents one of the first applications of discrete mathematics and dynamic network analysis to evaluate the long-term outcomes of Fotona laser therapy for OAB and introduces an innovative perspective for clinical decision-making in urogynecology.

## Introduction

Dynamic network analysis (DNA) is an emerging field that combines network theory and temporal analysis to study complex and evolving systems. This approach has been successfully applied in various domains by analyzing stock market dynamics to track information flow in engineering design projects [1,2]. DNA allows researchers to capture and analyze how network structures evolve over time, providing insights into the underlying mechanisms of complex systems.

In recent years, there has been growing interest in applying DNA to medical research, particularly in understanding disease progression and treatment efficacy [3]. The ability to model and visualize changes in patient symptoms and outcomes over time offers a powerful tool for clinicians and researchers. By treating patients as nodes in a network and their symptom similarities or treatment responses as edges, DNA can reveal patterns and trends that may be missed by traditional statistical analyses.

One area where DNA shows promise is the study of chronic conditions that require long-term management, such as overactive bladder (OAB) syndrome and urinary incontinence (UI). These conditions significantly impact the quality of life of millions of people worldwide, and their treatment often involves ongoing interventions and monitoring [4]. Recently, laser therapies such as the Fotona laser treatment (Fotona d.o.o., Ljubljana, Slovenia), which includes Vaginal Erbium Laser (VEL) and Urethral Erbium Laser (UEL), have emerged as promising alternatives to traditional treatments [5,6].

The efficacy of Fotona laser treatments (VEL + UEL) for OAB and UI is known to change dynamically over time, with patients showing varying responses at different post-treatment stages [7,8]. This temporal variability in treatment outcomes makes OAB and UI ideal candidates for DNA analysis. By applying DNA to longitudinal data from patients undergoing Fotona laser treatment, we can gain a deeper understanding of how these therapies affect symptom progression over time and identify patient subgroups that may respond more favorably to treatment.

In this technical report, we present a novel application of DNA to evaluate the longitudinal effects of Fotona laser therapy (VEL + UEL) on the OAB symptom score (OABSS) across multiple time points. The OABSS is a validated questionnaire that quantifies the severity of OAB symptoms on a scale of 0 to 15. Our approach integrates clustering techniques with network analysis to identify patterns of symptom improvement and to assess treatment outcome stability within distinct patient groups. This method builds upon recent advancements in the application of discrete mathematics and graph theory to urinary incontinence treatment decisions [9,10]. By visualizing these dynamic networks, we aim to provide clinicians with a powerful tool for personalizing treatment strategies and predicting long-term outcomes in patients with OAB and UI, potentially uncovering distinct subtypes of response patterns similar to those identified in related conditions [11]. 

## Technical report

Overview of the proposed analytical approach 

We present a novel analytical approach that integrates DNA with cluster analysis and principal component analysis (PCA) to analyze symptom change patterns in patients undergoing VEL + UEL laser therapy for OAB. This approach leverages established network analysis techniques while adapting them to the specific context of longitudinal clinical data [1]. The primary advantage of this method is its ability to represent complex longitudinal data in an intuitively understandable form, thereby capturing the dynamic nature of treatment responses over time. Our aim was to evaluate the effectiveness of VEL + UEL treatment across different patient groups and to identify subgroups with varying treatment responses.

The following Python code (Python Software Foundation, Wilmington, Delaware, United States) was developed and executed in Google Colab (Google LLC, Mountain View, California, United States) to perform DNA. This code implements the core functionality of the proposed analytical approach.

import pandas as pd

sklearn_preprocessing = importlib.import_module('sklearn.' + 'preprocessing')

sklearn_cluster = importlib.import_module('sklearn.' + 'cluster')

sklearn_decomposition = importlib.import_module('sklearn.' + 'decomposition')

# Load and preprocess the data

data = pd.read_csv('data.csv')

scaler = StandardScaler()

data_scaled = scaler.fit_transform(data[['T0-OABSS', 'T6-OABSS', 'T12-OABSS', 'T18-OABSS', 'T24-OABSS']])

# Perform KMeans clustering and PCA

kmeans = KMeans(n_clusters=3, random_state=42).fit(data_scaled)

pca = PCA(n_components=2).fit_transform(data_scaled)

# Example of visualizing the results (PCA plot)

plt.scatter(pca[:, 0], pca[:, 1], c=kmeans.labels_)

plt.xlabel('PCA Component 1')

plt.ylabel('PCA Component 2')

plt.show()

Application to VEL+UEL treatment data

This single-center, retrospective, before-and-after study was conducted between April 1, 2020, and March 31, 2022. It was approved by the Ethical Review Board of Yokosuka Urogynaecology and Urology Clinic (approval number: 24-C008).

Initially, 145 patients with OAB and pelvic organ prolapse (stage 2 or below) were enrolled. Of these, 102 had complete OABSS records over the two-year period, and 101 women aged ≥ 60 years provided final consent for data analysis. The inclusion criteria encompassed informed consent, inadequate improvement with pelvic floor muscle training (PFMT), absence of urogenital anomalies and pelvic malignancies, and no prior urethral operations. Exclusion criteria included withdrawal during opt-out, incomplete follow-up, and cognitive issues that prevented questionnaire completion. All participants underwent PFMT and received OAB medication for one year. The VEL+UEL treatment followed the protocol detailed in earlier studies by Okui and Okui [12] and Okui [13]. OAB severity was evaluated using the OABSS, with scores ranging from 0 to 15 (mild: <5, moderate: 6-11, severe: ≥12). The OABSS questionnaire consists of four questions: daytime frequency (0-2 points), nighttime frequency (0-3 points), urgency (0-5 points), and urgency incontinence (0-5 points). The total score is the sum of these four items. The primary endpoint was OABSS variation, assessed at baseline (T0) and 6 (T6), 12 (T12), 18 (T18), and 24 (T24) months post-intervention, with secondary endpoints including patient-reported UI symptoms and quality of life outcomes.

Detailed description of analytical methods

Cluster Analysis and Dynamic Network Construction

We computed a distance matrix between patients using standardized OABSS, employing Euclidean distance as the similarity metric. Hierarchical clustering using Ward's method was applied to categorize the patients based on their symptom profiles. The optimal number of clusters was determined using the elbow method, after which K-means clustering allocates patients to specific clusters, following which we constructed dynamic networks for each time point (T0-T24), where nodes represented individual patients and edges represented the strength of interpatient relationships based on symptom similarity. This approach allowed us to visualize how patient groups evolve over time, similar to the method used in the analysis of stock market dynamics [1].

Dimensionality reduction using PCA

PCA was applied to the standardized OABSS data to reduce the dimensionality of the dataset, facilitating a clearer interpretation of the clusters and their temporal evolution.

import pandas as pd

sklearn_cluster = importlib.import_module('sklearn.' + 'cluster')

sklearn_decomposition = importlib.import_module('sklearn.' + 'decomposition')

import matplotlib.pyplot as plt

# Load the data (assuming the data is in an Excel file named 'data.xlsx')

 data = pd.read_excel('data.xlsx')

# Select the columns to use

 columns_to_use = ['T0-OABSS', 'T6-OABSS', 'T12-OABSS', 'T18-OABSS', 'T24-OABSS']

 data_for_clustering = data[columns_to_use]

# Apply KMeans clustering

 kmeans = KMeans(n_clusters=3, random_state=42)

 clusters = kmeans.fit_predict(data_for_clustering)

# Apply PCA

 pca = PCA(n_components=2)

 pca_result = pca.fit_transform(data_for_clustering)

# Plot the PCA results

 plt.figure(figsize=(10, 7), dpi=300)

 colors = ['red', 'blue', 'green'] # Colors for the clusters

 for i in range(3):

 plt.scatter(pca_result[clusters == i, 0　], pca_result[clusters == i, 1　],

 color=colors[i], s=50, label=f'Cluster {i+1}')

 plt.xlabel('PCA Component 1')

 plt.ylabel('PCA Component 2')

 plt.legend()

 plt.show()

Based on the analysis results using the elbow method to optimize the number of clusters, the appropriate number of clusters was determined to be three. The elbow method involves plotting the sum of squared errors (SSE) within clusters as a function of the number of clusters and identifying the "elbow" point where SSE decreases sharply. In this case, the plot showed a significant decrease in SSE when the number of clusters was increased from two to three, after which the rate of decrease slowed down. This indicates that the three clusters are optimal for the dataset, as further increases in the number of clusters resulted in only marginal improvements in the SSE. 

**Figure 1:**
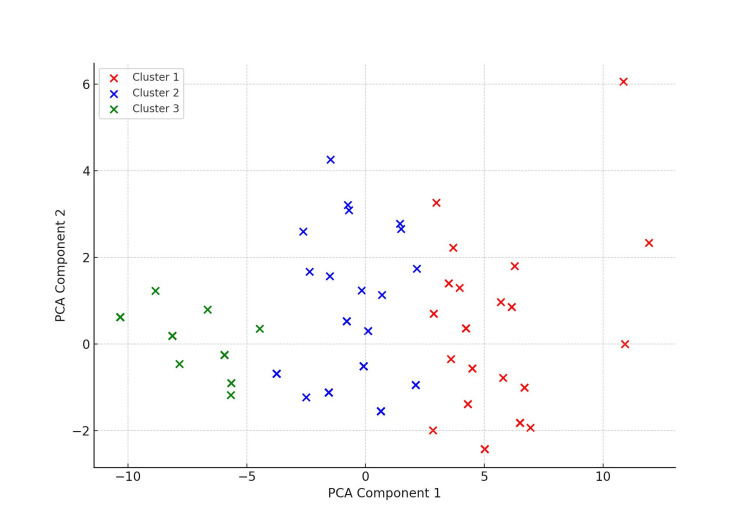
PCA Plot of OABSS Data with Three Clusters The vertical axis is Principal Component 2, and the horizontal axis is Principal Component 1. Both are unitless. Green points represent Cluster 0, red points represent Cluster 1, and blue points represent Cluster 2. Some data points overlap, making it appear as though there are fewer samples than there actually are: Cluster 0 has 34 samples, Cluster 1 has 47, and Cluster 2 has 20. PCA: Principal Component Analysis; OABSS: Overactive Bladder Symptom Score

Figure 1 shows the distribution of samples across these three clusters, where the green points represent Cluster 0, the red points represent Cluster 1, and the blue points represent Cluster 2. While the sample points appear to be fewer in number owing to significant overlap, the actual number of samples was as follows: Cluster 0 contained 34 samples, Cluster 1 contained 47 samples, and Cluster 2 contained 20 samples.

Network graphs 

We employed network graph techniques to quantify and visualize the changes in network structure over time. Edge lengths were calculated using the following formula: (inverse of the correlation coefficient - 1) × 1000, with Pearson's correlation coefficients quantifying relationships between patients' symptom scores across all time points. This approach was inspired by the work of Parraguez et al. on information flow in complex engineering projects [2]. The implementation of this analysis involved several steps, including data preprocessing, clustering, PCA, and network graph construction for each time point. The code demonstrates the process of loading the data, standardizing it, performing hierarchical clustering, applying K-means clustering, and creating dynamic network graphs for each time point (T0, T6, T12, T18, and T24). 

Our implementation began with the standardization of the OABSS data using StandardScaler (Scikit-learn Developers, Paris, France), followed by hierarchical clustering to determine the optimal number of clusters. We then applied K-means clustering to assign patients to clusters and performed PCA for dimensionality reduction and visualization. The final step involved creating dynamic network graphs for each time point, allowing us to observe the evolution of patient relationships over the course of treatment. 

**Figure 2:**
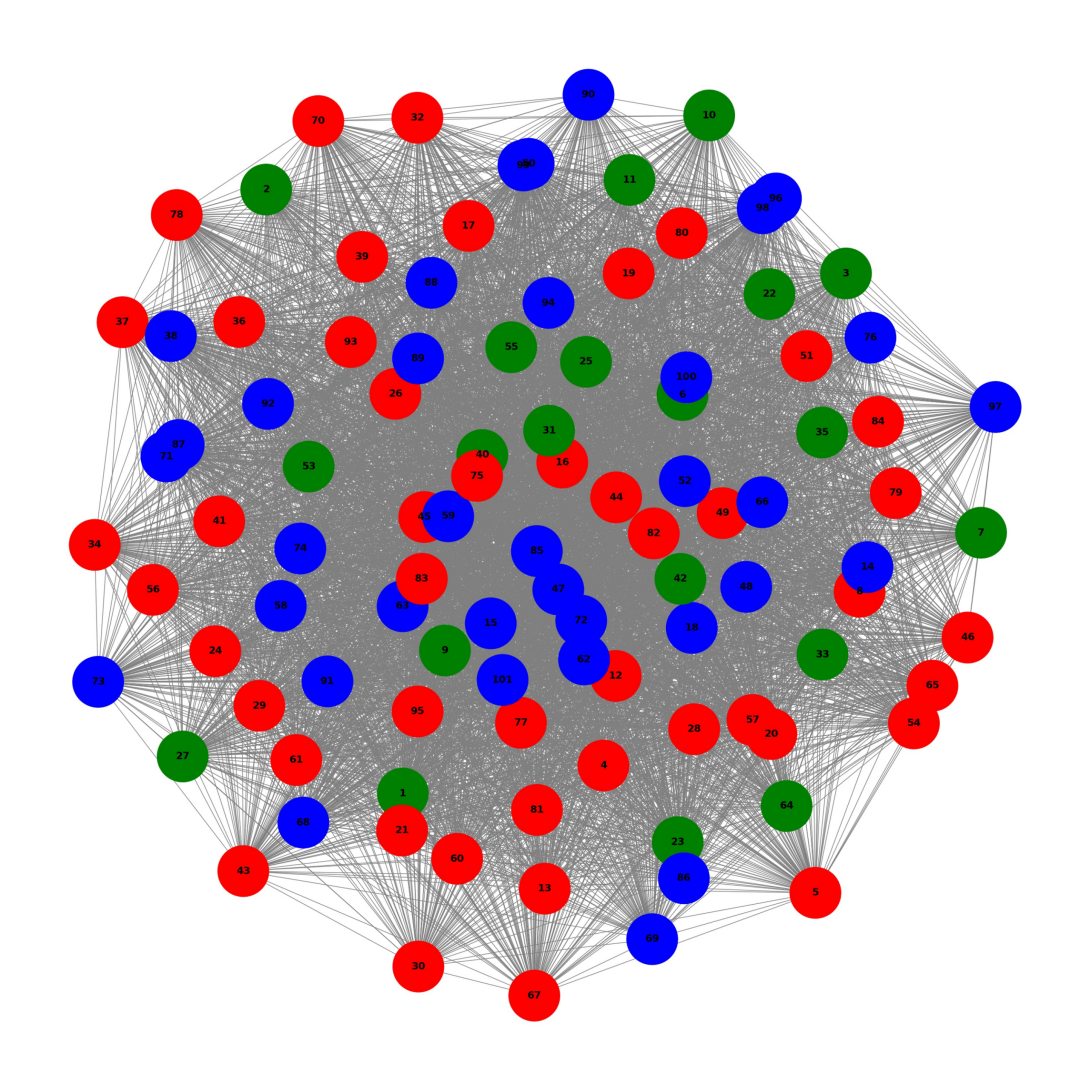
Network Graph at T0 Network graph at the baseline time point (T0) before the initiation of VEL+UEL laser therapy for overactive bladder (OAB) syndrome. Each node represents an individual patient, and edges indicate the similarity in symptom scores as measured by the OAB symptom score (OABSS). Node colors correspond to the patient clusters identified through hierarchical clustering: red for Cluster 1, blue for Cluster 0, and green for Cluster 2. The density of the network reflects the degree of symptom similarity among patients, with shorter edges indicating stronger correlations. This baseline network provides a visual representation of the relationships and groupings among patients prior to treatment.

Figure 2 shows the network graph at T0 for the three identified clusters. In this graph, nodes represent individual patients, with node colors corresponding to their respective clusters (green for Cluster 0, red for Cluster 1, and blue for Cluster 2). The edges between nodes represent the similarity between patients' OABSS scores, with edge lengths accurately reflecting the strength of the correlation between patients. The distribution of nodes shows that all three clusters are intermixed throughout the network without clear spatial separation. This suggests that, at baseline, patients from different clusters had similar symptom patterns, indicating a heterogeneous initial state across the study population.

This visualization allowed us to observe the initial structure of patient relationships and how they were grouped based on their symptom profiles at the beginning of the study. The subsequent time points (T6, T12, T18, and T24) show how these relationships and groupings evolve dynamically over the course of treatment.

**Figure 3:**
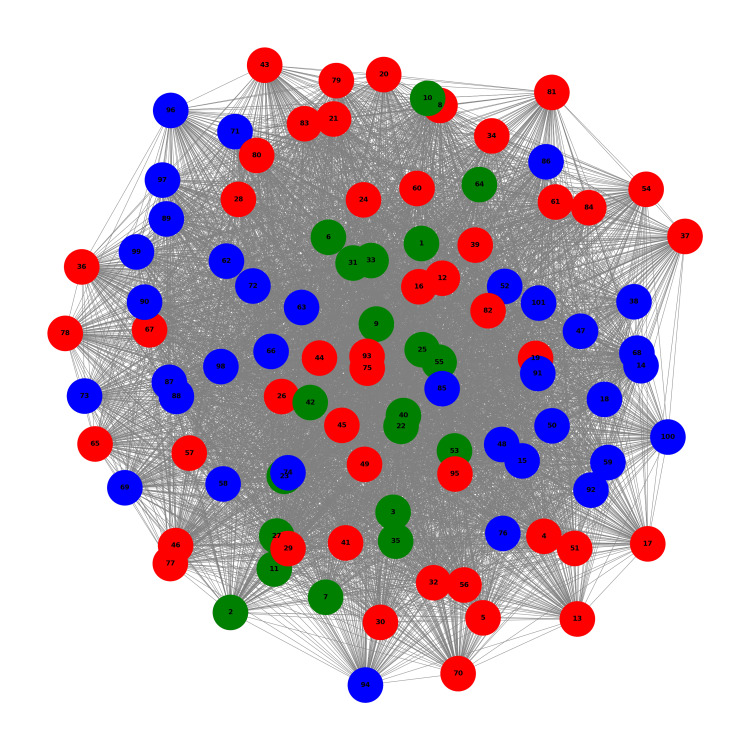
Network Graph at T6 Network graph at T6, six months after the initiation of VEL+UEL laser therapy for Overactive Bladder (OAB) syndrome. Node colors represent clusters as at T0: red for Cluster 1, blue for Cluster 0, and green for Cluster 2. The edges and density reflect the similarity in OAB symptom score (OABSS), with shorter edges indicating stronger correlations.

Figure 3 shows the network graph at T6, six months after the initiation of VEL + UEL laser therapy. The most notable change from the baseline (T0) was the apparent movement of Cluster 0 (green nodes) towards the center of the network. This visual shift suggests a potential change in the symptom profiles or treatment responses of the patients in this cluster. While the red and blue clusters maintained a relatively dispersed distribution similar to T0, the green cluster's centralization might indicate a more uniform response to treatment among these patients. This observation is particularly interesting because it provides a visual cue that aligns with the centrality analysis, which will be discussed in detail later. The overall network structure still shows intermixing of all three clusters, but with this distinct positional change in Cluster 0.

**Figure 4:**
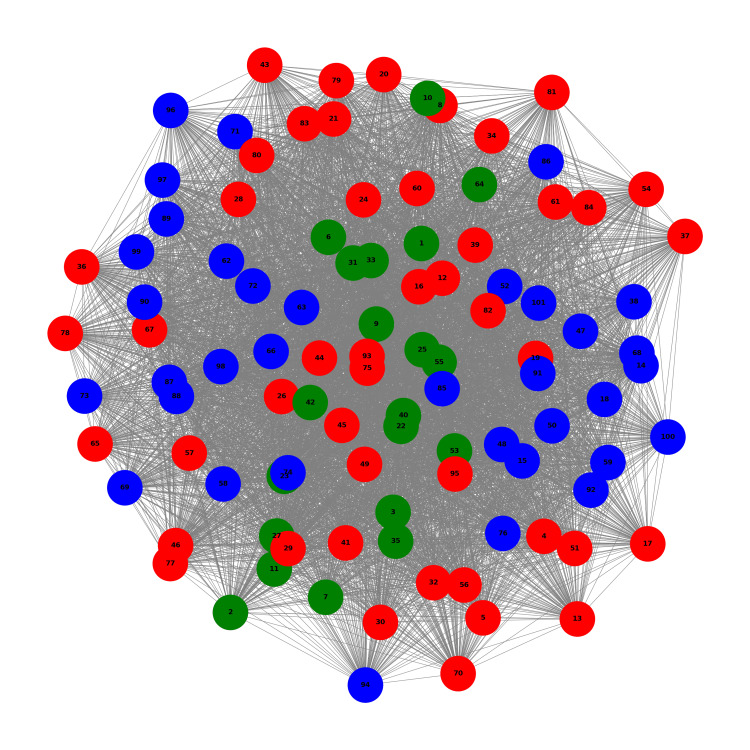
Network Graph at T12 Network graph at T12, 12 months after the initiation of VEL+UEL laser therapy for overactive bladder (OAB) syndrome. Node colors represent clusters as at T0: red for Cluster 1, blue for Cluster 0, and green for Cluster 2. The edges and density reflect the similarity in OAB symptom score (OABSS), with shorter edges indicating stronger correlations.

Figure 4 presents the network graph at T12 and 12 months after the initiation of VEL + UEL laser therapy. Visual inspection of this network revealed two notable changes from previous time points. First, cluster 0 (green nodes) continues to maintain its central position within the network, reinforcing the trend observed at T6. Second, and perhaps more strikingly, Cluster 2 (blue nodes) appears to have shifted towards the center as well. This visual impression suggests that patients in both green and blue clusters may experience similar changes in their symptom profiles or treatment responses. The red cluster (Cluster 1) remained more dispersed throughout the network. These observations, based on human visual perception, provide an intuitive understanding of the evolution of the network, which will be quantitatively analyzed later through centrality measurements. The apparent centralization of the two clusters at this time point suggests potentially divergent treatment outcomes among the three patient groups.

**Figure 5:**
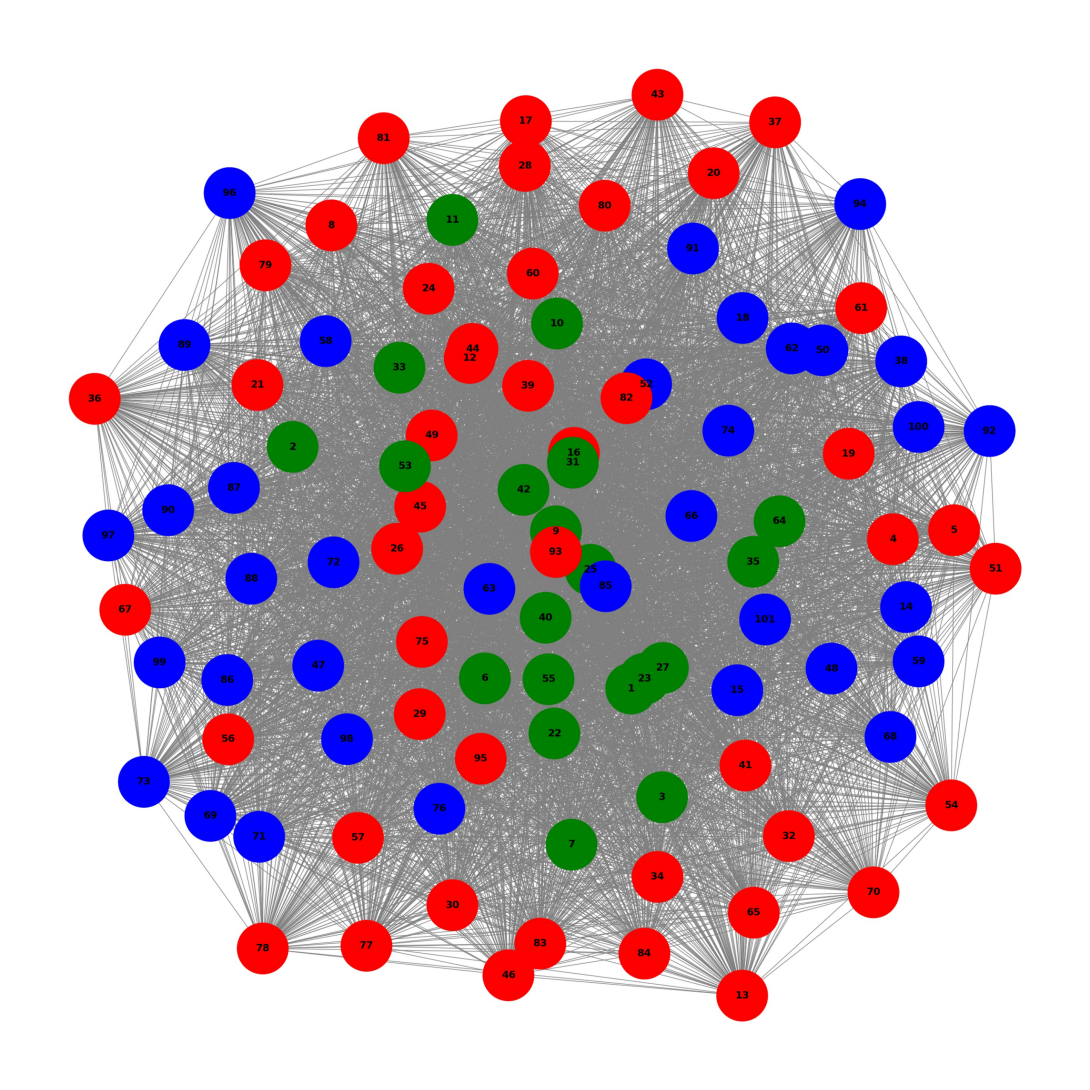
Network Graph at T18 Network graph at T18, 18 months after the initiation of VEL+UEL laser therapy for overactive bladder (OAB) syndrome. Node colors represent clusters as at T0: red for Cluster 1, blue for Cluster 0, and green for Cluster 2. The edges and density reflect the similarity in OAB symptom score (OABSS), with shorter edges indicating stronger correlations.

Figure 5 illustrates the network graph at T18, 18 months after the initiation of VEL + UEL laser therapy. Visual inspection of this network revealed several noteworthy changes from previous time points. Cluster 0 (green nodes) continues to maintain its central position within the network, a trend consistently observed since T6. Interestingly, Cluster 2 (blue nodes) appears to have shifted away from the center, in contrast to its position at T12. Perhaps the most striking change is the movement of Cluster 1 (red nodes) towards the center of the network, a pattern not observed at earlier time points. These visual observations suggest dynamic changes in the symptom profiles or treatment responses among the three patient groups over time. The apparent centralization of the green and red clusters coupled with the peripheral shift of the blue cluster at this time point suggests potentially divergent long-term treatment outcomes. These intuitive visual impressions provide a foundation for the subsequent quantitative analysis of cluster centrality, which offers a more precise understanding of these evolving network dynamics.

**Figure 6:**
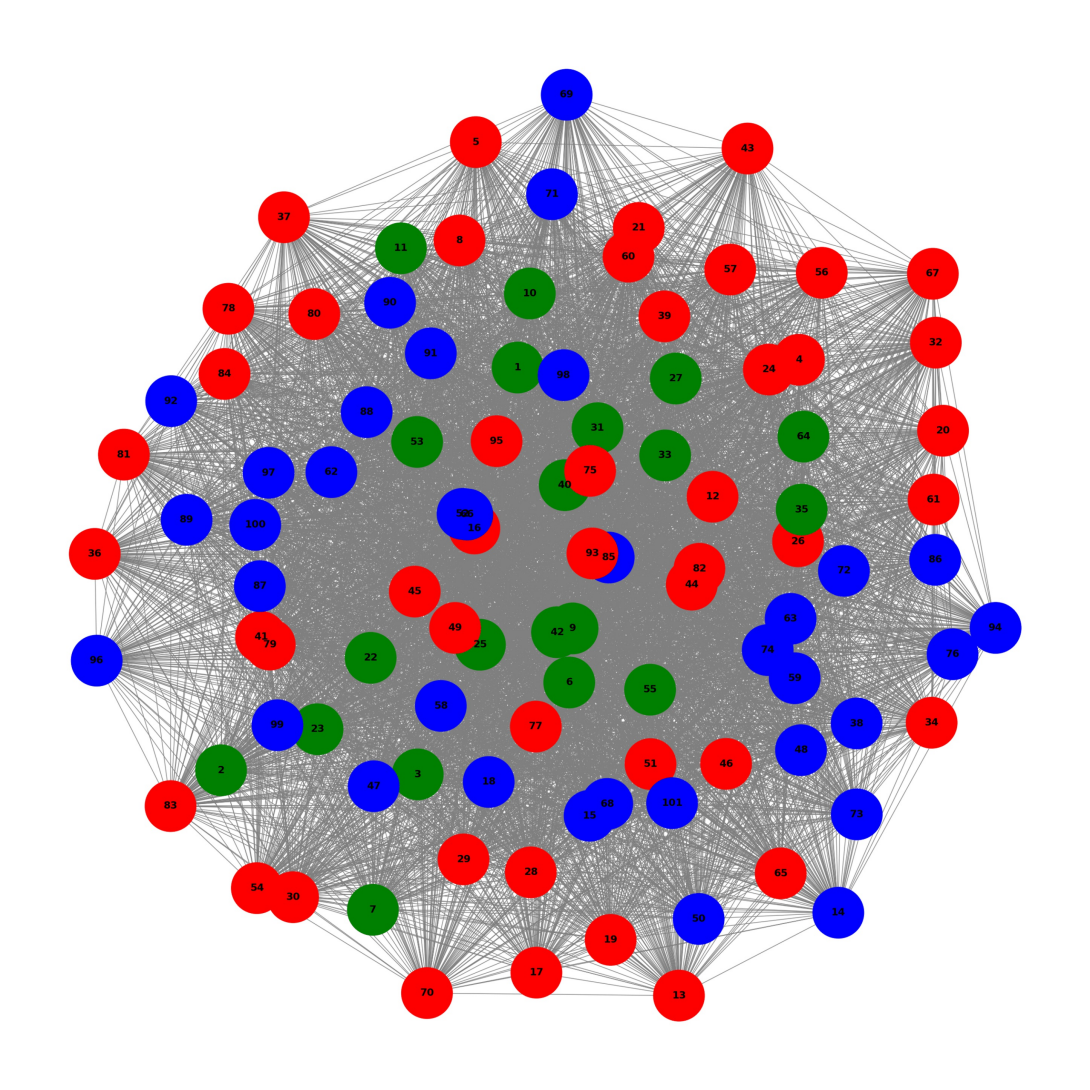
Network Graph at T24 Network graph at T24, 24 months after the initiation of VEL+UEL laser therapy for overactive bladder (OAB) syndrome. Node colors represent clusters as at T0: red for Cluster 1, blue for Cluster 0, and green for Cluster 2. The edges and density reflect the similarity in OAB symptom score (OABSS), with shorter edges indicating stronger correlations.

Figure 6 shows a network graph at 24 months (T24) after the initiation of VEL + UEL laser therapy. Visual inspection of this network revealed several interesting changes from previous time points. Cluster 0 (green nodes), while still maintaining a relatively central position, appears to have spread out considerably compared to earlier time points. This spread might suggest an increasing diversity in symptom profiles or treatment responses within this group. Clusters 1 (red nodes) and 2 (blue nodes), which were never fully centralized in the earlier stages, seemed to have maintained their previous positions without significant changes. Notably, Cluster 2 (blue) demonstrated a spread similar to that observed at T0, potentially indicating a return to baseline characteristics for this group, suggesting a complex evolution of patient groups over the course of treatment. The persistent central tendency of the green cluster, despite its spread, and the stable positions of the red and blue clusters, indicate distinct long-term treatment outcomes for each group. These intuitive visual impressions provide a foundation for the subsequent quantitative analysis of cluster centrality, which offers a more precise understanding of these evolving network dynamics and how they relate to treatment efficacy over time.

Dynamic analysis

**Figure 7:**
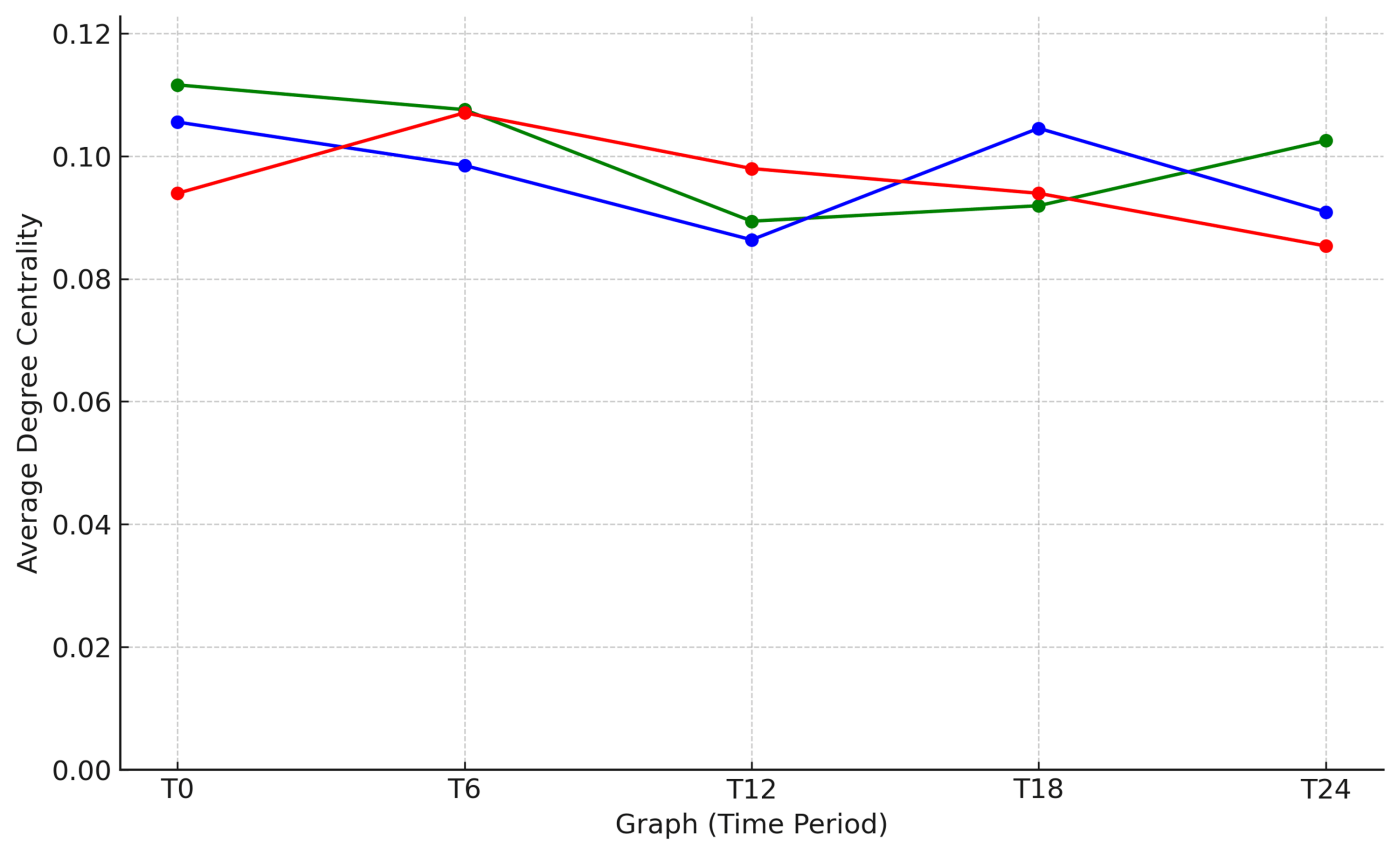
Dynamic Changes in Average Degree Centrality Across Three Clusters Dynamic changes in average degree centrality for three clusters (green, blue, red) over five time periods. The vertical axis represents the average degree centrality (unitless), and the horizontal axis shows the time periods (T0, T6, T12, T18, T24), where T0 is the initial time point, and subsequent labels represent later intervals.

Figure 7 illustrates the changes in the average degree centrality for the three main clusters (green, blue, and red) within the network at different time points (T0, T6, T12, T18, and T24) using DNA. This graph captures how each cluster evolves and diverges over time, providing a visual representation of its separation into distinct groups. In the graph, the green cluster maintains relatively stable centrality across all time points, with a slight increase over time. On the other hand, the blue cluster starts with relatively high centrality but gradually decreases over time. In contrast, the red cluster initially shows low centrality but experiences a sharp increase after T6, eventually reaching a centrality comparable to the other clusters. These trends suggest that the three clusters evolved into separate entities within the network over time. Changes in centrality reflect how each cluster is building and rebuilding its relationships with other nodes and clusters, providing crucial insights into the dynamics and evolution of the network.

To generate this figure, Python was used to calculate and plot the average degree centrality for each cluster. The following is an example of the code used to create the graph.

import matplotlib.pyplot as plt

import networkx as nx

import matplotlib.pyplot as plt

import numpy as np

from PIL import Image

import io

# Function to simulate network extraction from an image (placeholder for actual implementation)

 def extract_graph_from_image(image_path):

# Simulating the extraction of a network graph from an image using a random graph

 G = nx.erdos_renyi_graph(100, 0.1)

# Access node attributes using an alternative approach

 for i, node in enumerate(list(G)):

　　if i < 33:

　　　　G._node[node]['color'] = 'green'

　　elif i < 66:

　　　　G._node[node]['color'] = 'blue'

　　else:

　　　　G._node[node]['color'] = 'red'

# File paths for the images representing different time points

file_paths = [

 "network_graph_T0_colored.png",

 "network_graph_T6_colored.png",

 "network_graph_T12_colored.png",

 "network_graph_T18_colored.png",

 "network_graph_T24_colored.png"

]

# Extracting the graphs from the images

 graphs = [extract_graph_from_image(fp) for fp in file_paths]

# Time points and cluster colors

 time_points = ['T0', 'T6', 'T12', 'T18', 'T24']

 cluster_colors = ['green', 'blue', 'red']

# Step 1: Calculate Degree Centrality and plot

 cluster_averages = {color: [] for color in cluster_colors}

 for G in graphs:

 degree_centrality_func = importlib.import_module('networkx.algorithms.centrality').degree_centrality

 degree_centrality = degree_centrality_func(G)

 get_nodes = getattr(G, 'nodes')

 nodes_data = get_nodes(data=True)

 cluster_nodes = [n for n, attr in dict(nodes_data).items() if attr['color'] == color]

 for color in cluster_colors:

 avg_centrality = np.mean([degree_centrality[n] for n in cluster_nodes])

 cluster_averages[color].append(avg_centrality)

 plt.figure(figsize=(10, 6))

 items = getattr(cluster_averages, 'items') for color, averages in items():

 plt.plot(time_points, averages, label=f'Cluster {color}', marker='o')

 plt.title('Average Degree Centrality over Time')

 plt.xlabel('Time Point')

 plt.ylabel('Average Degree Centrality')

 plt.legend()

 plt.grid(True)

 plt.show()

# Step 2: Calculate Edge Density and plot

 def calculate_edge_density(G, cluster_nodes):

 subgraph = G.subgraph(cluster_nodes)

 possible_edges = len(cluster_nodes) * (len(cluster_nodes) - 1) / 2

 if possible_edges > 0:

 return subgraph.number_of_edges() / possible_edges

 else:

 return 0

 cluster_densities = {color: [] for color in cluster_colors}

 for G in graphs:

 for color in cluster_colors:

 get_nodes = getattr(G, 'nodes')

 nodes_data = get_nodes(data=True)

 cluster_nodes = [n for n, attr in nodes_data if attr['color'] == color]

 density = calculate_edge_density(G, cluster_nodes)

 cluster_densities[color].append(density)

 plt.figure(figsize=(10, 6))

 items = getattr(cluster_densities, 'items') for color, densities in items():

 plt.plot(time_points, densities, label=f'Cluster {color}', marker='o')

 plt.title('Edge Density over Time')

 plt.xlabel('Time Point')

 plt.ylabel('Edge Density')

 plt.legend()

 plt.grid(True)

 plt.show()

Changes in OABSS

In this study, we observed changes in the OABSS at various time points using T0 as the baseline. The results are summarized in Table 1, which presents the mean changes and standard deviations for each OABSS component and the total score at T6, T12, T18, and T24.

**Table 1:** Changes in OABSS Scores Over Time Changes in OABSS components (Q1 to Q4) and the total score over four time points (T6, T12, T18, T24) compared to the baseline (T0). T6, T12, T18, and T24 represent 6, 12, 18, and 24 months after the baseline, respectively. Values are presented as mean ± standard deviation, reflecting the average change from baseline at each time point. Q1: daytime frequency, Q2: nighttime frequency, Q3: urgency, Q4: urgency incontinence; OABSS: overactive bladder symptom score

Time Point	Change in Q1 (mean ± SD)	Change in Q2 (mean ± SD)	Change in Q3 (mean ± SD)	Change in Q4 (mean ± SD)	Change in Total Score (mean ± SD)	
T6	-0.0198 ± 0.1400	-0.0990 ± 0.3874	-0.4950 ± 0.8902	-1.0198 ± 1.0861	-1.6337 ± 2.0333	
T12	-0.0198 ± 0.1400	-0.0990 ± 0.3874	-0.4950 ± 0.8902	-1.0198 ± 1.0861	-1.6337 ± 2.0333	
T18	-0.0198 ± 0.1400	-0.0594 ± 0.4432	-0.4950 ± 0.8902	-0.9901 ± 1.1269	-1.5644 ± 2.1186	
T24	0.0000 ± 0.1414	-0.0396 ± 0.3980	-0.2871 ± 0.7660	-0.5050 ± 1.0452	-0.8317 ± 1.9394	

The OABSS consists of four questions assessing the key OAB symptoms: Q1 (daytime frequency), Q2 (nighttime frequency), Q3 (urgency), and Q4 (urgency incontinence). Each component is scored individually, with higher scores indicating more severe symptoms. The total OABSS is the sum of these four components, providing a comprehensive measure of OAB severity.

The data in Table 1 demonstrate changes in OABSS scores over time, with varying patterns observed across different components and time points. At T6 and T12, we observed modest improvements across all components, with the total score decreasing by 1.63 ± 2.03. Improvements in Q3 (urgency) and Q4 (urgency incontinence) were particularly notable at these time points. By T18, the improvements in Q2 (nighttime frequency) and Q4 showed slight variations, whereas Q1 and Q3 remained stable. Interestingly, at T24, we observed a slight increase in Q1 scores, while improvements in other components were less pronounced compared with earlier time points. The total score at T24 showed a decrease of 0.83 ± 1.94 from baseline, indicating a more modest long-term effect than initially suggested.

These results indicate that treatment led to initial improvements in symptoms, particularly urgency and urgency incontinence. These improvements were observed in the absence of any adverse effects. However, the long-term effects appear to be more complex, with some symptoms showing potential regression towards baseline levels. This pattern suggests that the treatment may have varying efficacies across different OAB symptoms and over time, highlighting the need for continued monitoring and possibly adjusted treatment strategies in the long-term management of OAB. 

## Discussion

DNA is a powerful tool in discrete mathematics for analyzing the temporal evolution of complex systems. Rooted in graph theory and matrix algebra, DNA provides a mathematical framework for representing and analyzing the structure and dynamic changes in networks [1]. This approach has gained significant traction in various social science fields because of its ability to capture and visualize intricate patterns of relationships and their evolution over time. In financial market analysis, Xu et al. utilized DNA to identify influential stocks, visualize relationships between stocks based on price fluctuation patterns, and identify central stocks that influence the overall market [1]. In engineering project management, Parraguez et al. applied DNA to analyze information flow in complex engineering design projects, enabling the identification of communication efficiency and critical information hubs at various project stages [2]. In the realm of public health, Valente employed DNA to study the role of social networks in the diffusion of health behaviors, contributing to the development of effective public health intervention strategies [3]. These diverse applications demonstrate the versatility and power of DNA to uncover hidden patterns and dynamics in complex social systems, providing insights that traditional analytical methods might overlook.

The application of DNA in this study revealed that patient responses to laser treatment were not uniform. The observation of different response patterns through cluster analysis suggests the need for an individualized approach to OAB treatment. The differences in responses between clusters indicate that various factors, potentially including patients' endocrine status, may influence treatment efficacy. Recent studies have shown associations between testosterone levels and OAB or stress UI (SUI) [14-16]. For instance, Li et al. [14] and Okui and Okui [15] found that testosterone levels were associated with the risk of OAB and SUI. However, as Giarenis et al. pointed out, high-quality evidence on the effectiveness of vaginal laser treatment is still lacking [16]. Future research should investigate the correlation between laser treatment effects and various patient factors, including hormone levels. Understanding the complex interrelationships between these factors will enable more precise individualized treatment approaches for OAB and SUI.

The mathematical foundation of DNA is primarily based on graph theory and matrix algebra. In graph theory, network structures are represented by nodes (vertices) and edges. In this study, patients were represented as nodes, and symptom similarities as edges. A graph G is defined as *G*=(*V, E*), where *V *is the set of nodes and *E* is the set of edges. Matrix algebra is employed to quantify network characteristics using adjacency matrices *A* and Laplacian matrices *L*. The adjacency matrix *A*, of size n × n, is defined such that *Aij *= 1, if nodes* i* and *j *are connected, and *Aij* = 0, if they are not. The Laplacian matrix *L* is given by *L=D−A*, where *D* is the degree matrix, containing the node degrees on its diagonal. Time series analysis is used to capture changes in the network structure over time through dynamic graphs and state-space models. The network state at time *t*, denoted* St*, represents the evolving condition of the network:

S_t​_=f(S_t−1​_)+ϵ_t​_

where f is the state transition function and ϵ_t_ is the noise term.

Clustering methods, such as k-means and hierarchical clustering, identify groups of nodes with similar characteristics. The objective function of k-means is defined as:

$J= \sum_{k}^{i= 1}\sum_{x\in C_i}^{}\|x - \mu_i \|^2$

where *k* is the number of clusters, *C_i_* is the iii-th cluster, and *μ_i​_*is the center of that cluster.

Node importance is evaluated using centrality measures, including degree centrality and eigenvector centrality. For instance, eigenvector centrality x satisfies the equation:

A_x_=λ_x_

where *λ* is the largest eigenvalue.

By combining these mathematical methods, DNA enables comprehensive analysis and visualization of complex system dynamics. In this study, these techniques were applied to evaluate the long-term effects of OAB treatment, revealing dynamic changes in patient groups that conventional statistical methods did not capture. This equation has been particularly supported by prior research in the calculation of centrality measures, such as eigenvector centrality, which is essential for understanding the influence and connectivity of nodes within the network [17]. Our approach extends this concept by incorporating dynamic changes in centrality over time, allowing for a more nuanced analysis of how individual nodes' importance evolves within a dynamic network.

For example, the change in average degree centrality over time for each cluster *C* is calculated as:

$Average Degree Centrality(C,t)= \frac{1}{\left|C \right|}\sum_{v\in C}^{}\frac{deg(v,t)}{\left|V \right|-1}$

where deg(v,t) is the degree of node vvv at time ttt, and ∣V∣ is the total number of nodes in the graph. This equation has been particularly supported by prior research in the calculation of centrality measures, such as eigenvector centrality, which is essential for understanding the influence and connectivity of nodes within the network [18]. Our approach extends this concept by incorporating dynamic changes in centrality over time, allowing for a more nuanced analysis of how individual nodes' importance evolves within a dynamic network.

This mathematical approach allows for detailed analysis of OAB treatment effects, capturing individual patients' temporal symptom changes as alterations in network structure. In conclusion, the application of DNA provides new perspectives on the individualization and optimization of OAB treatment, offering significant implications for future clinical research and treatment strategy development.

## Conclusions

This study applied DNA to evaluate the long-term efficacy of Fotona laser therapy for OAB syndrome, revealing distinct patient subgroups with varying treatment responses over a 24-month period. Our analysis identified three clusters with unique centrality evolution patterns: one maintaining stable centrality, another showing a gradual decrease, and a third experiencing a sharp increase after the initial treatment. These patterns correlated with changes in OABSS scores, particularly in the urgency and urgency incontinence components, which showed initial improvement followed by partial regression at 24 months. This novel approach not only quantified the heterogeneity in treatment responses but also demonstrated the potential for personalized OAB management strategies based on network dynamics. Future research should focus on correlating these network patterns with clinical and physiological factors to further refine the treatment protocols and improve long-term outcomes in patients with OAB.
